# Experimentation of the Bilinear Elastic Behavior of Plain-Woven GFRP Composite with Embedded SMA Wires

**DOI:** 10.3390/polym11030405

**Published:** 2019-03-01

**Authors:** Zhiwei Sun, Yingjie Xu, Wenzhi Wang

**Affiliations:** 1 Research Institute of Unmanned Aerial Vehicle, Northwestern Polytechnical University, Xi’an 710072, China; zhiwei@nwpu.edu.cn; 2 State IJR Center of Aerospace Design and Additive Manufacturing, Northwestern Polytechnical University, Xi’an 710072, China; 3 Shaanxi Engineering Laboratory of Aerospace Structure Design and Application, Northwestern Polytechnical University, Xi’an 710072, China; 4 School of Astronautics, Northwestern Polytechnical University, Xi’an 710072, China; wangwenzhi@nwpu.edu.cn

**Keywords:** shape memory alloy, GFRP composite, plain-woven composite, experiment, elastic behavior

## Abstract

In this paper, a plain-woven glass-fabric-reinforced polymer (GFRP) composite with embedded shape memory alloy (SMA) wires is investigated by means of experiments. The vacuum-assisted resin injection (VARI) method is utilized to fabricate the composite specimens. Quasi-static uniaxial tensile tests are then carried out to evaluate the influence of SMA reinforcement on the stress–strain behavior of the composite. Only the elastic behavior of the composite is considered in the present study. The tensile strain in all the experiments is kept below 2.5% to avoid debonding of the SMA-resin interface, which would lead to failure of the composite. Stress–strain curves are obtained and shown to present a bilinear behavior due to phase transformation taking place in the SMA wires beyond a certain stress threshold.

## 1. Introduction

The need for materials with ever-increasing performance in engineering applications has necessitated the development of multifunctional composites. An example of such a composite is obtained by embedding shape memory alloy (SMA) elements in the form of wires, particles, or ribbons into a matrix material. The unique behavior of SMAs, namely their superelasticity and shape memory effect, endows the composite with active abilities, including tunable stiffness, shape control, and self-healing. Moreover, the high energy density required for affecting phase transformations in shape memory alloys and their ability to deform extensively before failing make them interesting for damping and impact-resistance applications.

Fiber-reinforced polymer (FRP) composites have been widely used in various structural applications over the past decades, due to their low weight and high specific stiffness and strength. Recently, SMA wires have become commercially available with diameters below 0.2 mm. These small diameters allow the direct integration of SMA wires into FRP composites without losing the structural integrity of the matrix material. The integration of SMA wires into FRP composites can result in many benefits, which include temperature-controlled actuation, increased damage resistance, and improved damping capacity. SMA-wire-reinforced FRP composites have, therefore, been the subject of intense investigation over the last ten years [1,2,3,4,5,6,7,8,9,10,11,12,13].

An important research effort has been dedicated to characterizing the actuation response of SMA-wire-reinforced FRP composites, resulting from the shape memory effect of the SMA reinforcement. For instance, Tsoi et al. [1] conducted a detailed investigation of the actuation response of a Kevlar-fiber-reinforced epoxy resin laminate with embedded SMA wires. The recovery stress generated by various activation temperatures and its influence on the stress–strain response of the composite were reported. Galiotis’s group [2,3] integrated SMA wires into a laminated aramid-fiber-reinforced epoxy composite plate. The internal compressive stresses generated in the composite by resistive activation of the wires considering different temperatures, wire alloy compositions, and pre-strain levels were experimentally investigated. A similar SMA wire/aramid fiber/epoxy resin system was studied by Michaud et al. [4] and Balta et al. [5], who determined the stresses and strains generated in the composite in terms of the activation temperature and duration for a variety of pre-strain levels, volume fractions, and composite configurations. Araújo et al. [6] performed the three-point bending tests of unidirectional carbon-fiber-reinforced epoxy resin composite with embedded SMA wire actuators. The influence of the SMA wire actuators on the deflection of the composite was demonstrated. Lei et al. [7] presented an experimental and numerical investigation of the macroscopic mechanical behavior of a laminated glass-fiber-reinforced epoxy composite with SMA wires subjected to quasi-static loading. Taheri et al. [8] experimentally characterized the behavior of a SMA-wire-reinforced glass/epoxy laminate subjected to static loading. The authors discussed the influence of the constituents (i.e., the host composite and SMA), the SMA volume fraction, and the temperature on the overall behavior of the composite. The buckling behavior of a laminated glass-fiber-reinforced epoxy composite beam with SMA wires was studied by Choi et al. [9]. The loaded beam was exposed to an elevated temperature resulting in a phase transformation within the SMA wires. Experimental results indicate that the buckling of laminated composites can be controlled by phase transformation in the wires.

Moreover, the impact behavior of FRP composites with embedded SMA wires was investigated through instrumented impact tests. The embedded SMA wires can absorb the energy of the impact through pseudo-elastic deformation and thus improve the impact resistance of the composite structure. An extensive review can be found in the work of Angiono et al. [10]. Specifically, Paine and Rogers [11] investigated the use of SMA wires to improve the resistance to low-velocity impact damage of composite laminates. A cross-ply lay-up of graphite-fiber-reinforced bismaleimide composite with SMA wires was studied. It was shown that the SMA wires could prevent complete perforation of the composite samples due to impact. Aurrekoetxea et al. [12] discussed the influence of SMA wires on the low-velocity impact behavior of carbon-fiber-reinforced poly (butylene terephthalate) composites. Experimental results showed that the SMA has a positive effect on the maximum absorbed energy. Meo et al. [13] investigated, experimentally, the mechanical response of a SMA hybrid thermoplastic composite subject to low-velocity impact. They found that the embedding of SMA reinforcement could improve the damage resistance and ductility of composite structures.

The above literature survey indicates that most of the experimental characterizations carried out so far have been dedicated primarily to FRP laminates or unidirectional composites with embedded SMAs. In contrast, research into FRP textile composites with SMA reinforcement is still insufficient at present, in both experimentation and modeling. The idea of combining the concepts of textile composites and SMA reinforcement seems promising. Firstly, the interlacing of fiber yarns of the woven or braiding fabrics in textile composites has several advantages, e.g., increasing the intra- and inter-laminar strength, greater damage tolerance, as well as providing a possibility to produce near-net-shape structural components. Secondly, the tremendous diversity of textile architecture allows various configurations for introducing SMA components.

The purpose of the present paper is to investigate plain-woven composites with embedded SMA wires experimentally. A plain-woven glass-fabric-reinforced polymer composite with embedded SMA wires (SMA/plain woven glass-fabric-reinforced polymer GFRP) is studied in this work. An important potential of hybridizing GFRP composites using SMA wires is to improve their impact resistance [10]. The embedded SMA wires are capable of absorbing the energy of the impact through pseudo-elastic deformation, reducing the effects of the impact on the composite structure. The vacuum-assisted resin injection method is used to fabricate plain-woven glass-fabric-reinforced 5105 AXSON epoxy resin composite samples with embedded SMA wires. To evaluate the influence of SMA reinforcement on the mechanical properties of the samples, uniaxial tensile tests were conducted, in which the maximum tensile strain was kept below 2.5% to avoid failure by debonding of the SMA-resin interface. According to [14], during the tensile test of SMA-wire-reinforced resin matrix composite, the interfacial debonding of SMA and resin would occur at a strain of about 2.5%. Therefore, only the elastic behavior of the composite is considered in this paper.

## 2. Materials and Work Method

### 2.1. Materials

The plain-woven glass fabrics are supplied by Guangzhou CABEN Composite Co., Ltd., China. The surface density of the plain-woven glass fabric is 200 g/m^2^. The polymer material is a 5015 AXSON epoxy resin and the hardening agent is EH208.

**Figure 1:**
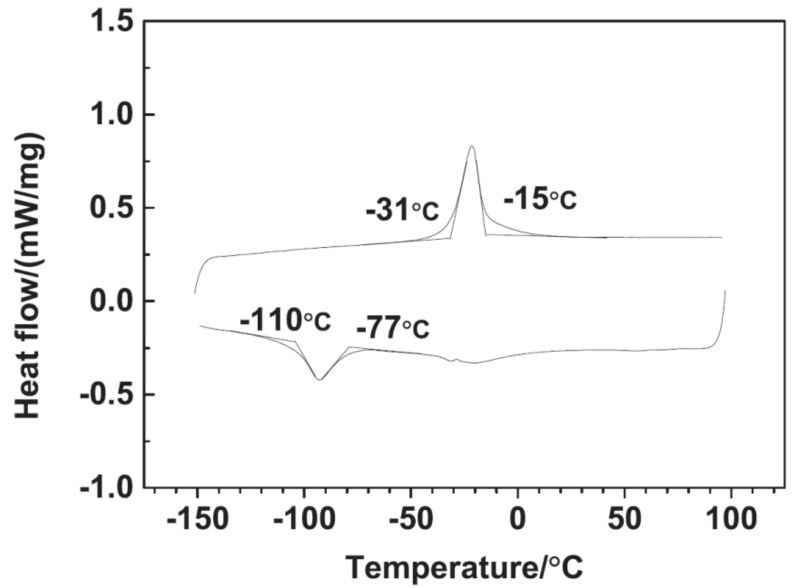
Differential scanning calorimeter (DSC) results of shape memory alloy (SMA).

The SMA wire used in this study is a NiTi alloy (50.8% Ni) wire produced by Xi’an Saite Metal Materials Co., Ltd., China. The diameter of the wire is 1 mm. The transformation temperatures of the SMA are measured using a differential scanning calorimeter (DSC). The results are plotted in Figure 1. The phase transformation temperatures are determined to be as follows: martensite start temperature $M_{s}\approx -110{}^{\circ}C$, martensite finish temperature $M_{f}\approx -70{}^{\circ}C$, austenite start temperature $A_{s}\approx -31{}^{\circ}C$, and austenite finish temperature $A_{f}\approx -15{}^{\circ}C$.

**Figure 2:**
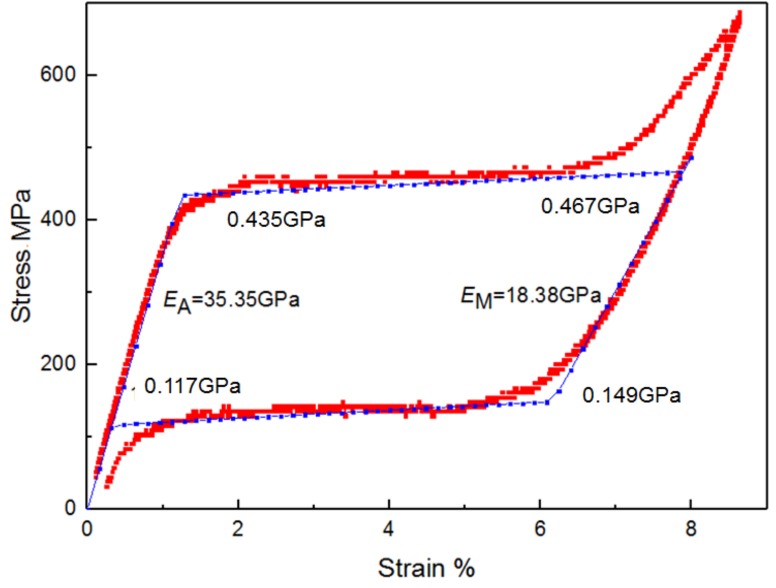
Stress–strain curve of a SMA wire subjected to a uniaxial loading cycle.

Moreover, the loading–unloading test at room temperature is implemented for a SMA wire to obtain the transformation stress values. Figure 2 shows the stress–strain curve of a SMA wire subjected to a uniaxial loading-unloading cycle at a loading rate of 3 mm/min. From the figure, it can be seen that the onset of the forward phase transformation from austenite to martensite corresponds to a martensite start stress $\sigma_{s}^{AM}$ = 434.66 MPa. The transformation completes when the stress reaches the martensite finish value $\sigma_{f}^{AM}$ = 467.10 MPa. During the unloading process, the reverse transformation from martensite to austenite starts at the austenite start stress $\sigma_{s}^{MA}$ = 149.21 MPa and finishes at the austenite finish value $\sigma_{f}^{MA}$ = 116.78 MPa. Note that the strain shown in Figure 2 is not completely recovered. The incomplete recovery is due to the formation of residual strains during loading. Perfect recovery can be achieved by training, which consists of first subjecting the samples to a series of loading cycles until stabilization of the residual strain. The Young’s modulus of pure austenite and martensite phases can also be determined from the curves. They are found to be *E*_A_ = 35.35 GPa and *E*_M_ = 18.38 GPa.

The properties of glass fiber and of the 5015 AXSON epoxy resin are provided by the producer and are given in Table 1.

### 2.2. Manufacturing

**Figure 3:**
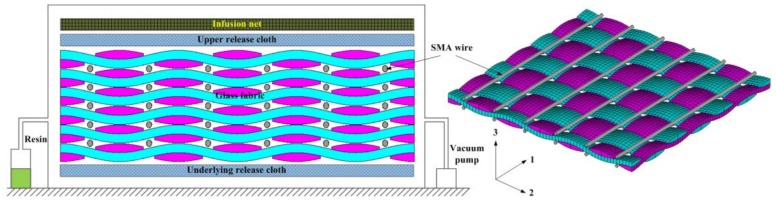
Illustration of the vacuum-assisted resin injection (VARI) installation and stacking pattern of the SMA wires and fabrics.

In this study, the SMA/plain-woven GFRP samples are manufactured by vacuum-assisted resin injection (VARI). Figure 3 shows the schematic diagram of the VARI installation together with the stacking pattern of SMA wires and glass fabrics. There are a total of six fabric layers in the laminate. Emery paper (600-grade) which is used to polish the SMA wires for better interfacial bonding with the resin. The polished SMA wires are placed between subsequent glass fabric layers and distributed along the length direction at uniform intervals, as shown in Figure 3.

**Figure 4:**
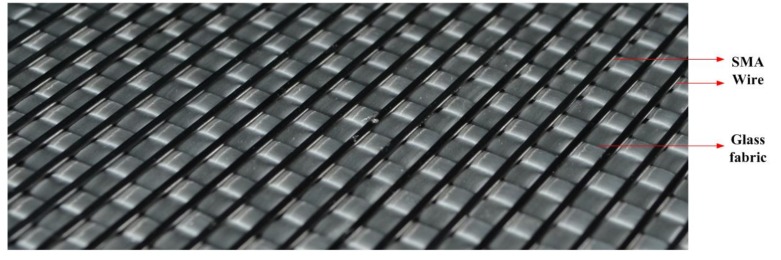
Picture of a glass fabric layer with SMA wires on top.

A square glass plate is placed at the bottom as a holder in the VARI process, and the samples are manufactured over it. The manufacturing process is organized as follows:

(1) A release cloth is evenly coated on the surface of the glass plate. One layer of glass fabric is placed on the release cloth, and the SMA wires are placed on top of the glass fabric layer. This process is repeated until all the glass fabrics and SMA wires are stacked according to the schematic diagram shown in Figure 3. A picture of a portion of a glass fabric layer with the SMA wires on top is shown in Figure 4.
(2) The stacked fabrics with embedded SMA wires are covered with the release cloth and infusion net, which is then closed by a vacuum bag and a sealant tape. To draw a vacuum inside the enclosed space and ensure the uniform flow of resin, two delivery pipes are fixed on both sides of the device. These two pipes are respectively connected to the vacuum pump and the resin infusion port.
(3) The resin is mixed with the hardening agent and accelerating agent with a mass ratio of 100:1.5:0.15. After ensuring that the device does not leak, the resin mixture is infused into the vacuum device through the infusion port. The whole system is cured at room temperature for 24 h at a vacuum level of 600 mbar.

Samples of dimensions 250 × 25 × 3 mm^3^ (American Society for Testing and Materials (ASTM) D3039/D3039M-08 [15]) are cut from the square laminates using a water jet cutting machine. Aluminum tabs of 75 mm length and 3 mm thickness are bonded to both ends of the specimens to prevent crushing of specimen during gripping. For comparison, plain-woven GFRP composite specimens without SMA wires are also fabricated. The manufacturing process and the geometry of the specimens are exactly the same as those of the SMA/plain-woven GFRP composite samples. Furthermore, the mass fractions of different components in the samples are listed in Table 2.

### 2.3. Tensile Test

**Figure 5:**
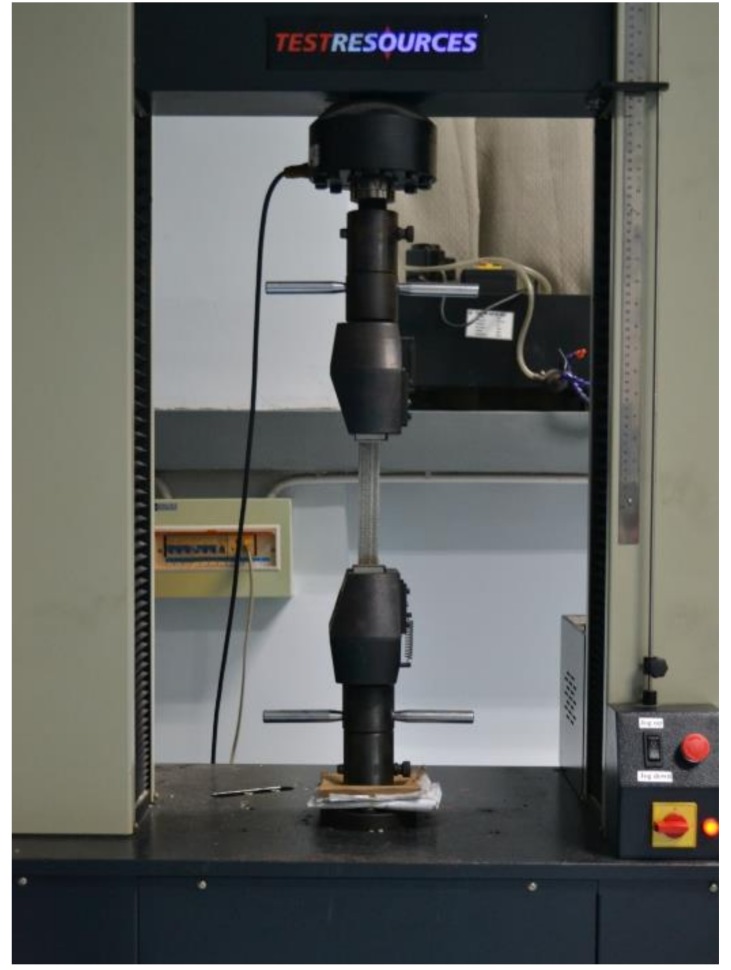
Experimental setup for the uniaxial tensile tests.

To evaluate the influence of SMA reinforcement on the mechanical behavior of the composite samples, uniaxial tensile tests are carried out on samples with and without SMA wires using a TEST RESOUCES electronic universal testing system, see Figure 5. Note that the sensor is removed to show the specimen clearly. All the tests are performed at room temperature with a 3 mm/min loading rate. A total of three samples are tested for each kind of material (SMA wires and GFRP composites with and without SMA wires).

## 3. Results and Discussion

**Figure 6:**
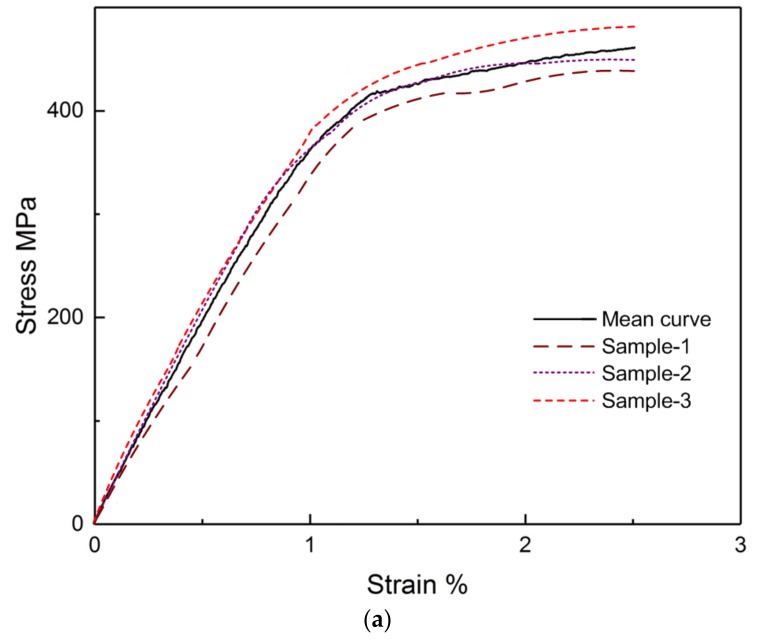

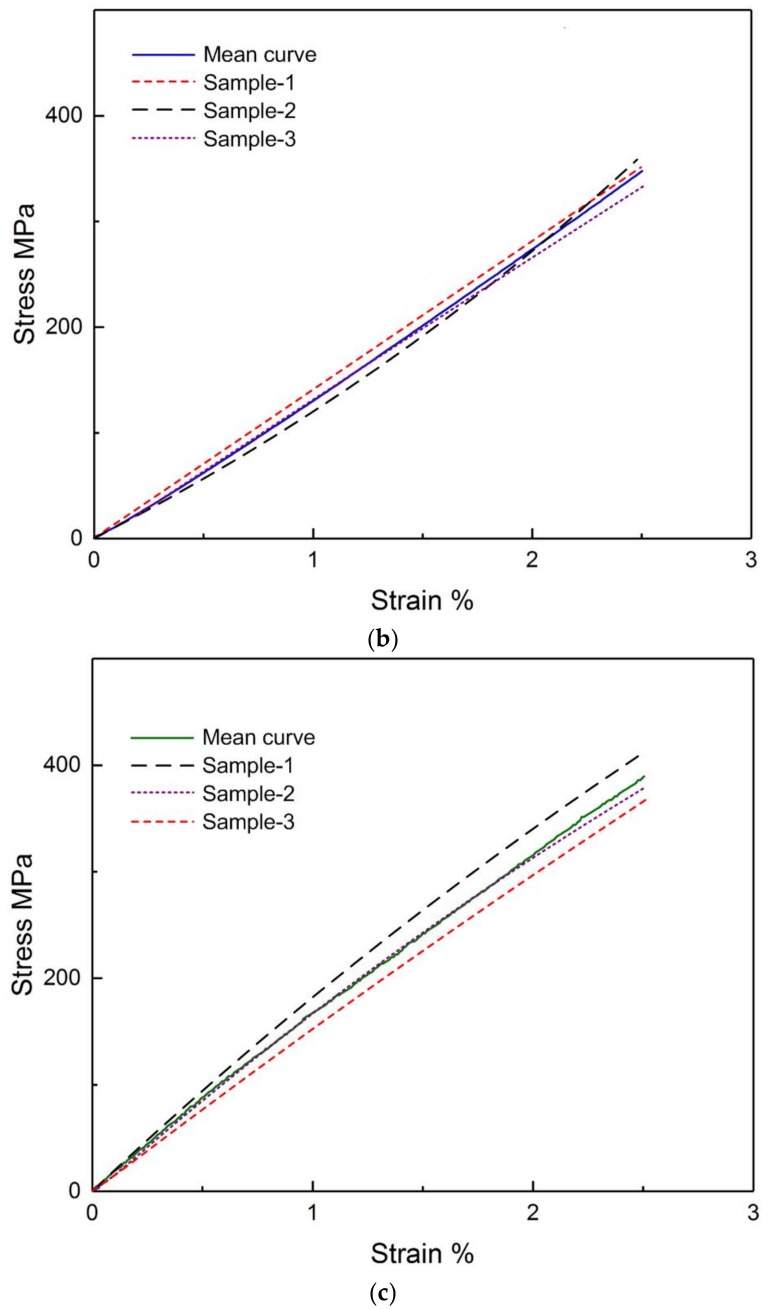
The experimental stress–strain curves for each kind of material: (**a**) SMA wire; (**b**) plain-woven GFRP composite; (**c**) SMA/plain-woven GFRP composite.

The experimental stress–strain curves of SMA wire, plain-woven GFRP, and SMA/plain-woven GFRP composites are presented in Figure 6, respectively. The mean curve for each kind of material is also indicated in the figures.

**Figure 7:**
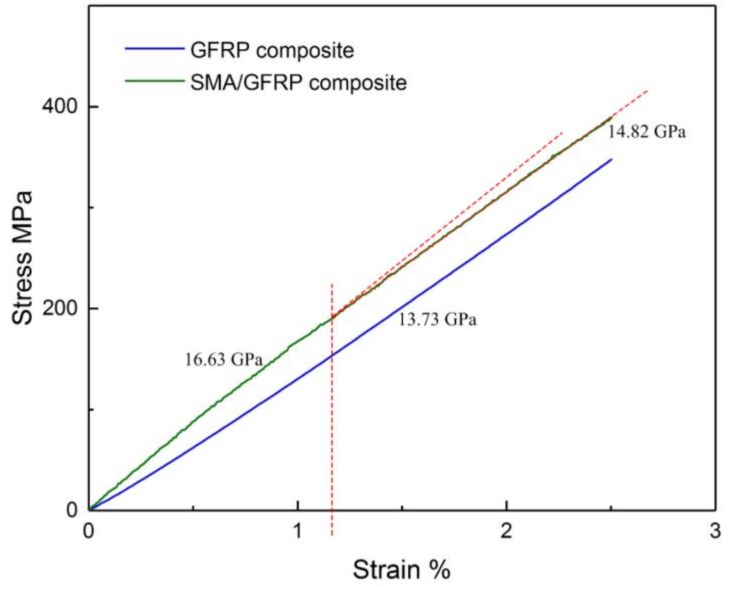
Stress–strain curves of plain-woven GFRP composites with and without SMA wires.

In order to investigate the effect of SMA wire, the stress–strain curves of plain-woven GFRP and SMA/plain-woven GFRP composites are further compared in Figure 7. The effect of SMA wire on the overall mechanical behavior of the composite is noticeable. It can be seen that the stress–strain curve of SMA/plain-woven GFRP composite appears to follow a bilinear relationship while the composite without SMA has a linear behavior. 

**Figure 8:**
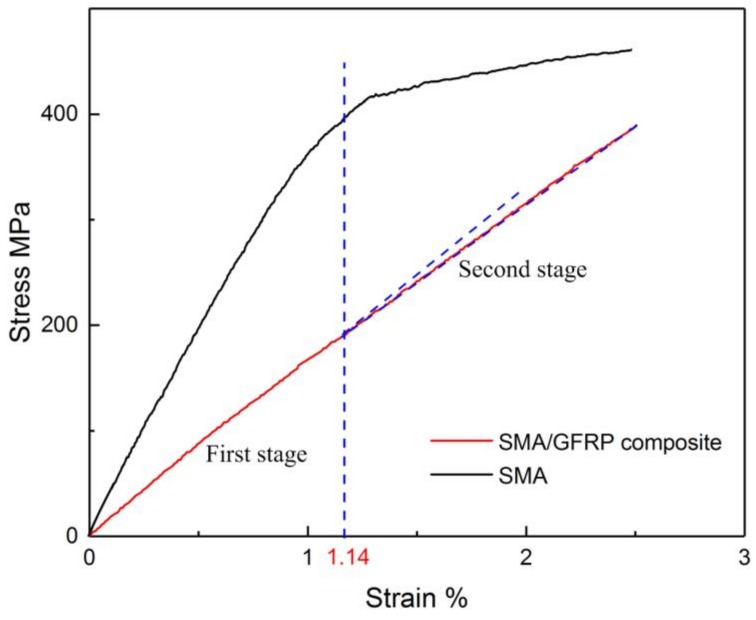
Stress–strain curves of SMA wire and SMA/plain-woven GFRP composite samples.

The bilinear stress–strain relationship of the SMA/plain-woven GFRP composite is the result of a phase transformation in the SMA, as illustrated in Figure 8, which leads to softening of the sample response. In the first stage of loading, as the axial strain increases from 0% to 1.14%, the SMA wire and the composite are both in the linear elastic domain. In the second stage, in which the strain increases beyond = 1.14%, the stress in the SMA exceeds the threshold for the forward phase transformation; the material then starts to transform from austenite to martensite. As a result, the overall behavior of the composite sample is affected.

Moreover, the effective modulus of the composite is significantly affected by the incorporation of SMA reinforcement. As seen in Figure 6, the Young’s modulus of the composite without SMA is 13.73 GPa while the Young’s modulus of SMA/plain-woven GFRP composite varies between 16.63 GPa during the first stage and 14.82 GPa during the second stage. 

## 4. Conclusion and Perspectives 

In this paper, an experimental investigation of the mechanical behavior of a SMA/plain-woven GFRP subjected to uniaxial tensile loading is presented. An experimental setup is proposed that allows the fabrication of SMA/plain-woven GFRP samples by means of a VARI process. Experimental results indicate that the stress–strain curve of the SMA/plain-woven GFRP composite follows a bilinear relationship, which is explained by the occurrence of a phase transformation within the embedded SMA wires.

Only the elastic behavior of the composite is considered here, with the tensile strain in all the experiments kept below 2.5% to avoid debonding of the SMA-matrix interface and failure of the composite. Thus, the interfacial debonding or composite failures are not considered in this paper. This aspect of the composite behavior will be the topic of future investigations. An important potential of hybridizing GFRP composite using SMA wires is to improve their impact resistance. Thus, the impact behavior of the SMA/plain-woven GFRP composite will be another important topic in future studies. Moreover, it is worth pointing out that various constitutive models have been presented in the literature to describe the mechanical behavior of SMA. These constitutive models can be further combined with the micromechanical approach to analyze the mechanical behavior of the composites with embedded SMA materials in an efficient way.

## Figures and Tables

**Table 1:** Properties of glass fiber and epoxy resin.

Constituent	Young’s Modulus (GPa)	Poison Ratio
Glass fiber	70.0	0.22
Epoxy resin	2.0	0.4

**Table 2:** The mass fractions of different components in the samples. GFRP: glass-fabric-reinforced polymer.

Samples	Glass Fiber (%)	SMA (%)	Resin (%)
GFRP sample	73.26	0	26.74
SMA/GFRP sample	68.35	7.92	23.73

## References

[1] Tsoi K.A., Schrooten J., Zheng Y., Stalmans R. (2004). Part II. Thermomechanical characteristics of shape memory alloy composites. Mater. Sci. Eng..

[2] Parthenios J., Psarras G., Galiotis C. (2001). Adaptive composites incorporating shape memory alloy wires. Part 2: development of internal recovery stresses as a function of activation temperature. Compos. Part A: Appl. Sci. Manuf..

[3] Bollas D., Pappas P., Parthenios J., Galiotis C. (2007). Stress generation by shape memory alloy wires embedded in polymer composites. Acta Materialia.

[4] Michaud V. (2004). Can shape memory alloy composites be smart?. Scr. Mater..

[5] Balta J.A., Michaud V.J., Parlinska M., Gotthard R., Manson J.A.E. Adaptive composite materials processing. Proceedings of the European conference on macromolecular physics, structure development upon polymer processing: physical aspects.

[6] De Araújo C.J., A Rodrigues L.F., Neto J.F.C., Reis R.P.B. (2008). Fabrication and static characterization of carbon-fiber-reinforced polymers with embedded NiTi shape memory wire actuators. Smart Mater. Struct..

[7] Lei H., Wang Z., Tong L., Zhou B., Fu J. (2013). Experimental and numerical investigation on the macroscopic mechanical behavior of shape memory alloy hybrid composite with weak interface. Compos. Struct..

[8] Taheri-Behrooz F., Taheri F., Hosseinzadeh R. (2011). Characterization of a shape memory alloy hybrid composite plate subject to static loading. Mater. Des..

[9] Choi S., Lee J.J., Seo D.C., Choi S.W. (1999). The active buckling control of laminated composite beams with embedded shape memory alloy wires. Compos. Struct..

[10] Angioni S.L., Meo M., Foreman A. (2010). Impact damage resistance and damage suppression properties of shape memory alloys in hybrid composites—A review. Smart Mater. Struct..

[11] Paine J.S., Rogers C.A. (1994). The response of SMA hybrid composite materials to low velocity impact. J. Intell. Mater. Syst. Struct..

[12] Aurrekoetxea J., Zurbitu J., De Mendibil I.O., Agirregomezkorta A., Sanchez-Soto M., Sarrionandia M. (2011). Effect of superelastic shape memory alloy wires on the impact behavior of carbon fiber reinforced in situ polymerized poly(butylene terephthalate) composites. Mater. Lett..

[13] Meo M., Marulo F., Guida M., Russo S. (2013). Shape memory alloy hybrid composites for improved impact properties for aeronautical applications. Compos. Struct..

[14] Lei H., Wang Z., Zhou B., Tong L., Wang X. (2012). Simulation and analysis of shape memory alloy fiber reinforced composite based on cohesive zone model. Mater. Des..

[15] ASTM D3039/D3039M-08 (2008). Standard Test Method for Tensile Properties of Polymer Matrix Composite Materials.

